# First person – Maria Losada-Perez

**DOI:** 10.1242/dmm.049067

**Published:** 2021-06-01

## Abstract

First Person is a series of interviews with the first authors of a selection of papers published in Disease Models & Mechanisms, helping early-career researchers promote themselves alongside their papers. Maria Losada-Perez is first author on ‘ A novel injury paradigm in the central nervous system of adult *Drosophila*: Molecular, cellular and functional aspects’, published in DMM. Maria is a postdoc in the lab of Sergio Casas-Tintó at the Cajal Institute (CSIC) in Madrid, Spain, investigating glial cell responses to central nervous system (CNS) injuries and, in this context, cell communication.


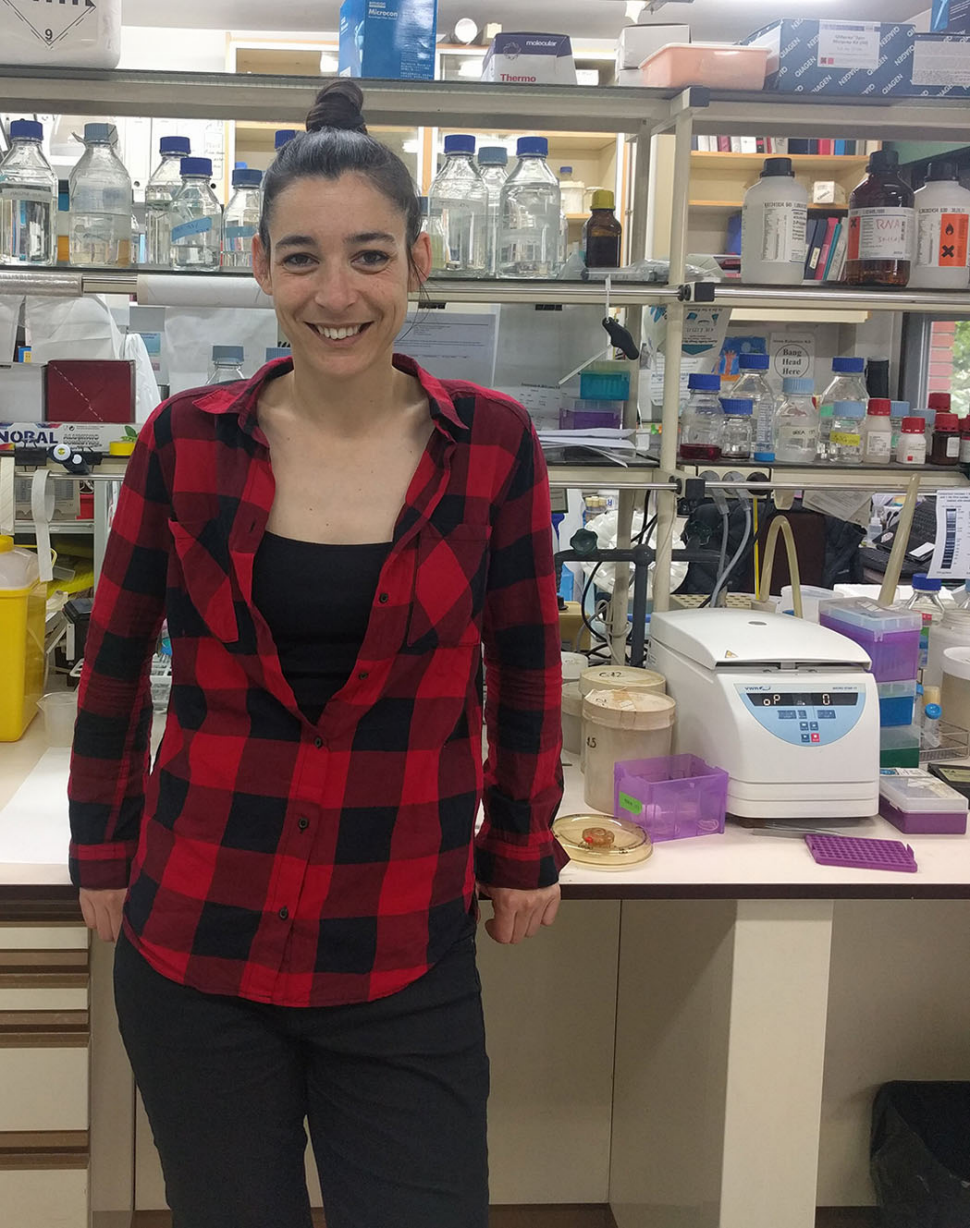


**Maria Losada-Perez**

“[…] we share 60% of our genome with fruit flies […].”

**How would you explain the main findings of your paper to non-scientific family and friends?**

The CNS in humans does not regenerate; therefore, lesions due to spinal cord injury, stroke or neurodegenerative diseases can result in permanent disabilities. We use the fruit fly *Drosophila melanogaster* to understand what is going on in the nervous system after injury. To do so, we have developed a method to generate ‘paralytic’ fruit flies. We found that these paralytic flies are able to recover mobility after a ‘spinal cord’ injury. In this paper, we describe some of the molecular mechanisms that elicit CNS recovery, and several genes that are required for it. This is important because we share 60% of our genome with fruit flies and, therefore, it is reasonable to think that the genes and pathways we discovered by using this animal model are of value regarding future regenerative medicine.

**What are the potential implications of these results for your field of research?**

This new injury method could be used in other laboratories to research cellular and systemic responses to CNS damage. We describe for the first time that adult fruit flies recover after an injury in the CNS, which opens the possibility of CNS regeneration in adult humans. The genes and pathways we have already linked with regeneration could be tested in other model organisms and are potential targets for future treatment – not only for spinal cord injuries but also for stroke and neurodegenerative diseases.

**What are the main advantages and drawbacks of the model system you have used as it relates to the disease you are investigating?**

The main advantage of *Drosophila* are its genetics. We can do almost whatever we imagine, although this is simultaneously a challenge and a great responsibility. Many people believe that the evolutionary distance between flies and humans is a drawback. However, I believe this is also an advantage because if the genes and mechanisms we discover by using *Drosophila* are conserved in humans, those genes and mechanisms are essential and, probably, more important than those that are more specific for mammals.

“[…] wild-type flies recover locomotion after CNS injury.”

**What has surprised you the most while conducting your research?**

That wild-type flies recover locomotion after CNS injury.

**Describe what you think is the most significant challenge impacting your research at this time and how will this be addressed over the next 10 years?**

Achieving CNS regeneration is the main goal of my research. I plan to use this injury paradigm to understand what is going on within the injury site, how glial cells detect the damage and the different mechanisms of healing it. I will also make use of next generation sequencing to identify key genes involved in this process.

**What changes do you think could improve the professional lives of early-career scientists?**

**Figure:**
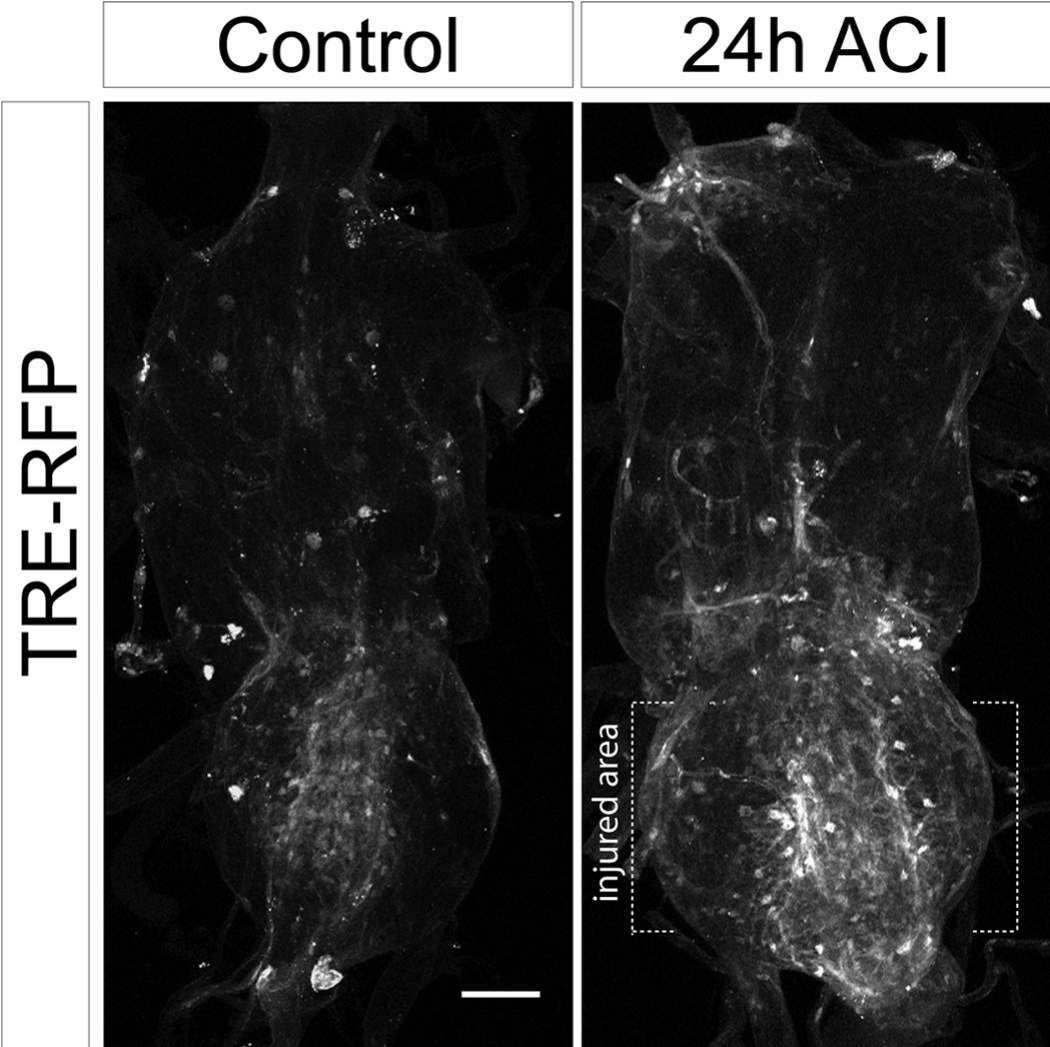
**JNK signalling pathway is activated upon crush injury**. Representative images of not injured and injured (24 h After Crush Injury, ACI) VNCs with the JNK reporter TRE-RFP.

I believe that governments should increase the science budget, as this would help us to focus on our research rather than regularly applying for fellowships.

**What's next for you?**

I would like to set up my own lab and use the technique we have developed, in order to study other aspects of CNS injury and regeneration. My main goal is to find key genes or targets that promote CNS regeneration.
